# Purification of Natural Pigments Violacein and Deoxyviolacein Produced by Fermentation Using *Yarrowia lipolytica*

**DOI:** 10.3390/molecules28114292

**Published:** 2023-05-24

**Authors:** Georgio Nemer, Nicolas Louka, Paul Rabiller Blandin, Richard G. Maroun, Eugène Vorobiev, Tristan Rossignol, Jean-Marc Nicaud, Erwann Guénin, Mohamed Koubaa

**Affiliations:** 1 Université de Technologie de Compiègne, ESCOM, TIMR (Integrated Transformations of Renewable Matter), Centre de Recherche Royallieu—CS 60319, 60203 Compiègne CEDEX, France; georgio.nemer@utc.fr (G.N.); rabillerpaul@gmail.com (P.R.B.); eugene.vorobiev@utc.fr (E.V.); erwann.guenin@utc.fr (E.G.); 2 Laboratoire CTA, UR TVA, Centre d’Analyses et de Recherche, Faculté des Sciences, Université Saint-Joseph, Beyrouth 1104 2020, Lebanon; nicolas.louka@usj.edu.lb (N.L.); richard.maroun@usj.edu.lb (R.G.M.); 3 Université Paris-Saclay, INRAE, AgroParisTech, Micalis Institute, 78350 Jouy-en-Josas, France; tristan.rossignol@inrae.fr (T.R.); jean-marc.nicaud@inrae.fr (J.-M.N.)

**Keywords:** *Yarrowia lipolytica*, pigment, violacein, deoxyviolacein, fermentation, purification, column chromatography

## Abstract

Violacein and deoxyviolacein are bis-indole pigments synthesized by a number of microorganisms. The present study describes the biosynthesis of a mixture of violacein and deoxyviolacein using a genetically modified *Y. lipolytica* strain as a production chassis, the subsequent extraction of the intracellular pigments, and ultimately their purification using column chromatography. The results show that the optimal separation between the pigments occurs using an ethyl acetate/cyclohexane mixture with different ratios, first 65:35 until both pigments were clearly visible and distinguishable, then 40:60 to create a noticeable separation between them and recover the deoxyviolacein, and finally 80:20, which allows the recovery of the violacein. The purified pigments were then analyzed by thin-layer chromatography and nuclear magnetic resonance.

## 1. Introduction

Microbial dyes and pigments have gained momentum during the last decade due to the detrimental effects of synthetic dyes and consumers’ aversion to their consumption [1]. As such, biosynthetic pigments, microbial or otherwise, have rightly benefited from increased interest [2], which has further fueled active research in genetic engineering, fermentation, and extraction technologies. Unlike the process that enables the synthesis of their synthetic counterparts, the process of producing microbial pigments as secondary metabolites has less direct negative impact on the environment, as it limits the industry’s dependence on petroleum extraction. In addition to aesthetic coloring properties that change the color of textiles and other surfaces, microbial dyes and pigments often possess functional antioxidant, or antitumor, and antimicrobial properties [3]. The latter of these properties could very well be exploited in efforts to create functional clothing (e.g., medical gowns). However, despite their interesting range of properties, certain natural dyes can be hazardous to human health and therefore need to be thoroughly researched before use [4]. Violacein is a bis-indole pigment synthesized as a secondary metabolite by a number of bacterial species, the most notable of which are *Janthinobacterium violaceum*, *Duganella violaceinigra*, and *Chromobacterium violaceum* [5,6,7,8]. Research suggests that violacein has a wide range of properties, with current literature suggesting it has reasonable efficacy as an antifungal [9,10], antitumoral [11], antipyretic [12], and antiviral agent [13]. Other research also suggests that it may have immunomodulatory properties [14] as well as suppressive and inhibitory activity against *Staphylococcus aureus* and other multidrug-resistant pathogens [15,16].

In microorganisms naturally capable of producing violacein, the pigment is produced by the enzymatic oxidation and condensation of two L-tryptophan molecules through the action of five enzymes, encoded by the genes VioA, VioB, VioC, VioD, and VioE [17]. This pathway also leads to the synthesis of deoxyviolacein, which, according to the available literature, seems always to accompany the synthesis of violacein, with the latter being the dominant component [18]. What is referred to in the literature as crude violacein is a mixed pigment output composed of violacein, deoxyviolacein, and derivatives from the same pathway. This metabolic pathway is well characterized; therefore, various heterologous production chassis have been considered for the safe and effective production of violacein. Most previous research seems to focus on conventional model organisms, with *Escherichia coli* at the center [19,20,21]. To a lesser extent, the nonconventional yeast *Yarrowia lipolytica* has been used for such production [22,23]. *Y. lipolytica*, a GRAS oleaginous yeast, is a versatile production chassis whose potential in this application among myriad others is bolstered by the availability of a large panoply of genetic toolkits enabling its genetic manipulation. To date, *Y. lipolytica* has been successfully modified to synthesize a wide range of fatty acids [24,25] and flavonoids, among other molecules of interest, which it produces as secondary metabolites [26,27]. Another unique feature of this microorganism is its ability to utilize numerous carbon sources including dextrose and glycerol. It has also been successfully modified to utilize and thus valorize a wide range of industrial wastes and effluents [28,29].

The production of violacein has been carried out on a small scale, although a number of studies have described the production of violacein at relatively larger bioreactor scales using conventional model organisms [20], *Janthinobacterium lividum* [30], *Citrobacter freundii* [18], and novel violacein-producing bacterial species isolated from diverse environments [31]. The present study describes the biosynthesis of a mixture of violacein and deoxyviolacein using a genetically modified *Y. lipolytica* strain as a production chassis, the subsequent extraction of the intracellular pigments, and ultimately their purification using column chromatography. This study is the first describing the optimization of solvents used for the separation of violacein and deoxyviolacein.

## 2. Results and Discussion

### 2.1. Pigment Recovery

**Figure 1:**
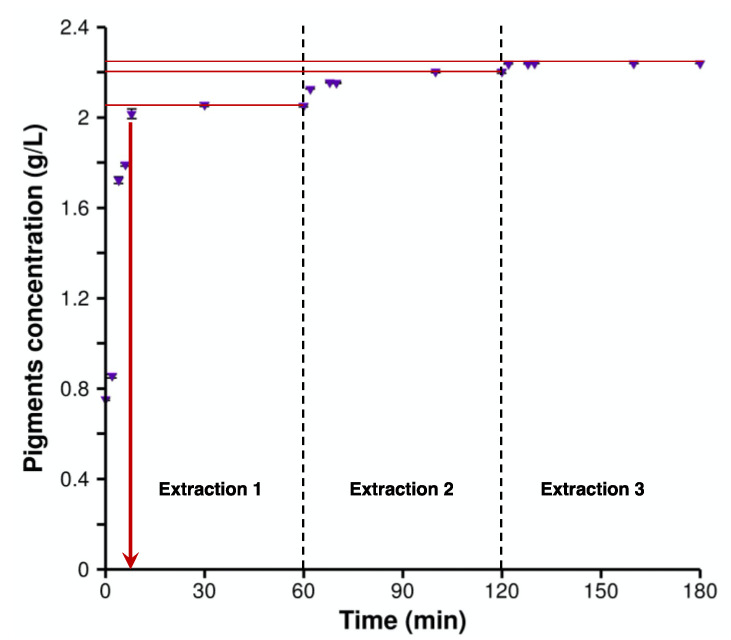
Time course of crude violacein extraction. Cumulative concentration (g/L) of pigments (violacein and deoxyviolacein) in ethanol 96% over time throughout three extractions (purple symbols). Red lines are given to guide the eye to show the extracted pigments after each extraction.

The pigments were recovered after fermentation in 96% ethanol, using a double-jacket vessel. The total mass of crude violacein produced by the *Y. lipolytica* within a 168-h was 300 mg, which represents 2% of the total weight of the recovered biomass. The extraction yield was evaluated by periodic sampling and quantification of the violacein. Ninety-two percent of the total amount of pigment was extracted in the first 8 min of the first extraction (Figure 1), with the modest remainder of the violacein extracted in the second extraction. In fact, the overall viscosity and relative cohesion of the biomass resulted in a slower dispersion of the cells into smaller clumps with a greater biomass/solvent contact surface.

After analyzing the pigment extract by HPTLC we found that the pigment-rich solvent resulting from the first extraction was mainly composed of deoxyviolacein, which accounted for 61.7% *v*/*v* of the pigment load. The result of the second extraction contained more violacein than deoxyviolacein, with violacein accounting for 55.7% of the total pigment load. The pigment load of the third extraction consisted exclusively of violacein. The 1:1:1 mixture contained approximately 50% violacein and 50% deoxyviolacein. The results of the first extraction do not agree with those reported in the literature, where crude violacein is composed mainly of violacein compared to deoxyviolacein [32]. The variance in the proportions of violacein/deoxyviolacein between the solvents resulting from all three extractions could indicate that deoxyviolacein has a greater affinity for ethanol than does violacein.

### 2.2. Optimization of Solvent Composition for Pigment Separation

**Figure 2:**
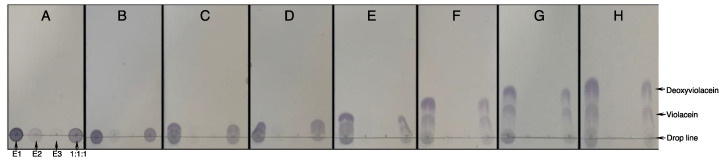
Determination of optimal solvent ratio for violacein/deoxyviolacein separation. Thin layer chromatography assays to evaluate the efficacy of the solvent mixtures considered. The sequence of pigment deposits, from left to right, is as follows: Extraction 1 (E1), Extraction 2 (E2), Extraction 3 (E3), 1:1:1 mixture. (**A**–**H**) correspond to the solvents’ ethyl acetate/cyclohexane ratio of 10:90, 20:80, 30:70, 40:60, 50:50, 60:40, 65:35, and 80:20, respectively.

In order to select the appropriate solvent for the separation of violacein and deoxy-violacein, several mixtures of organic solvents were evaluated, including cyclohexane, ethanol, methanol, ethyl acetate, and acetonitrile, with or without the addition of triethylamine. The best compromise was obtained with a mixture of cyclohexane and ethyl acetate, of which different compositions were tested. The results of the TLC migration assays are summarized in Figure 2. Solvent “G” showed an adequate capacity to separate both pigments without causing significant trailing and thus pigment mixing as observed with solvent “H”. Solvent “D” appeared to elute mainly deoxyviolacein, the less polar pigment, as evidenced by the migrating purple spot and the static blue spot. Therefore, alternating between solvents “D” and “G”, ethyl acetate/cyclohexane, (40:60) and ethyl acetate/cyclohexane (65:35), respectively, was considered as part of the purification protocol.

By comparing different solvent compositions, it can be seen that a higher polar fraction of the solvent produces a more pronounced migration. Therefore, cyclohexane was considered as a wetting agent for the silica 60 stationary phase.

### 2.3. Separation of Violacein and Deoxyviolacein Using Column Chromatography

Once the appropriate solvents for pigment separation were selected, violacein and deoxyviolacein were separated by column chromatography. The 1:1:1 configuration was chosen as the separation sample because it would be more representative of a 1:30 *w*/*v* biomass-to-solvent ratio. In addition, optimizing an appropriate method for this configuration would eliminate the need to develop methods adapted to the yield of each extraction. Using the same column, larger amounts of either solvent would result in larger bands that would not be adequately separated and resolved over the length of the column.

**Figure 3:**
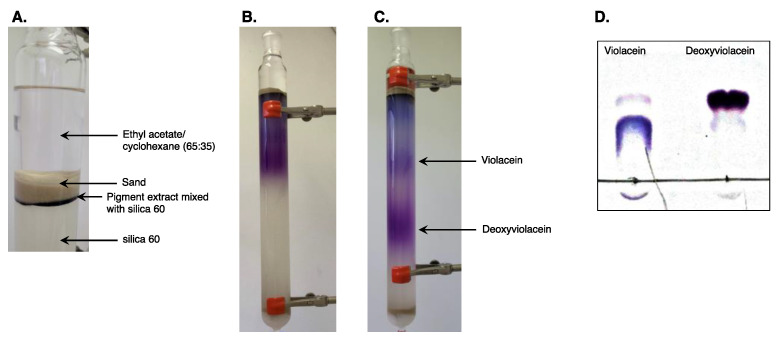
(**A**) Chromatography column load with pigment extract. (**B**) Solvent “G” was added until two distinct colors were observed at the extremities, with violacein at the top and deoxyviolacein at the bottom. (**C**) Solvent “D” was added until the deoxyviolacein was sufficiently distant from the violacein. (**D**) Purified extracts were analyzed on a TLC plate.

Solvent configuration “G” has the ability to separate both compounds and provide sufficiently distinct spots. In addition, this solvent combination had a relatively low affinity for lipophilic compounds, making it suitable for the purification of a complex matrix containing the lipids also produced by *Y. lipolytica* as secondary metabolites in addition to cell debris. The stationary phase consisted of cyclohexane-saturated silica 60, which has a remarkably low affinity for violacein. A 4 cm × 50 cm column (SEVAL, Poggibonsi, Italy) was used. The bottom of the column was coated with a small amount of cotton covered with a thin layer of fine sand. Then 300 g of silica wetted with cyclohexane was poured into the column and compacted by carefully draining the solvent. After pouring and subsequent adequate compaction of the silica by draining the solvent, 200 mg of analyte was added and then wrapped in a layer of fine sand to minimize the risk of disruption (Figure 3A).

Solvent “G” was first used until both pigments were clearly visible and distinguishable (Figure 3B). To create a noticeable separation between them, solvent “G” was substituted with solvent “D”, which favored the migration of the deoxyviolacein and a virtual stasis of the violacein band (Figure 3C). Upon recovery of the deoxyviolacein, the solvent “D” was replaced by solvent “H” until the violacein component of the pigment load was recovered. The recovered pigment load was then dried by evaporating the solvent under reduced pressure and analyzed using a TLC plate (Figure 3D). The results show that the violacein and deoxyviolacein were well purified, with one major band of each of pigment (traces of deoxyviolacein were observed in the violacein fraction, and vice versa).

### 2.4. NMR Spectroscopy Analysis of Pigments

**Figure 4:**
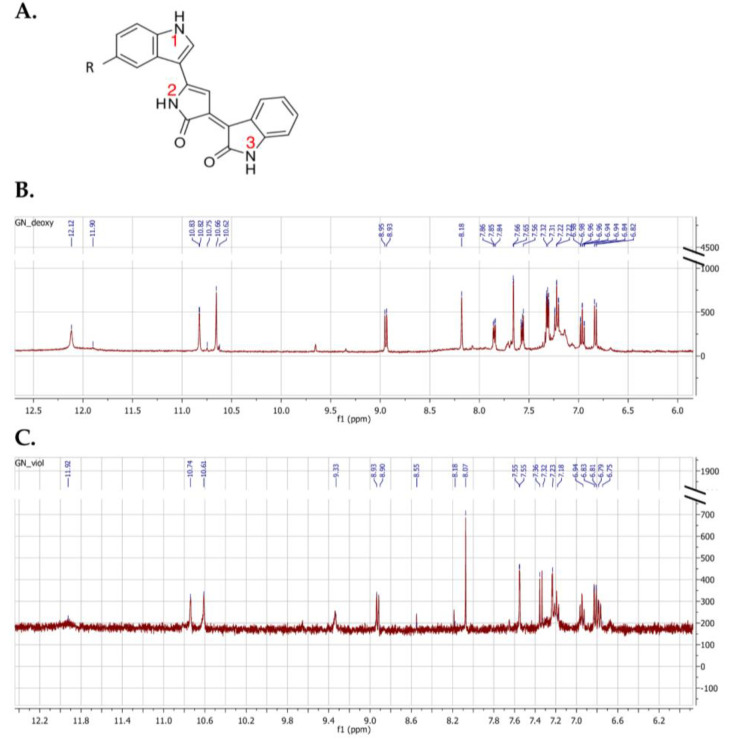
(**A**) Common molecular scaffold of violacein and deoxyviolacein with numbers assigned to each amide and indolic proton. For violacein, R would be a hydroxyl group; for deoxyviolacein, R would be hydrogen. (**B**) ^1^H-NMR spectrogram for deoxyviolacein. (**C**) ^1^H-NMR spectrogram for violacein.

In order to identify the pigments and confirm their purity, data on the indolic amine group and the two amides were evaluated using NMR spectroscopy. For the sake of simplicity, numbers were assigned to each of the amide protons (2 and 3) and to the indolic N-H proton (1) contained in the molecule, and the chemical shifts detected by ^1^H-NMR associated with each of them were compared with the values reported in the literature. Figure 4A shows the common molecular structure of violacein and deoxyviolacein. In the case of violacein, R would be a hydroxyl group whereas in the case of deoxyviolacein, R would be hydrogen.

The results are in agreement with those reported by Wille and Steglich [33] regarding the chemical synthesis of violacein and deoxyviolacein (Figure 4B,C and Table 1), which are a benchmark for numerous studies aimed at identifying and purifying these pigments [34,35,36]. The chemical shift in the indolic proton (1) appears to be clearly different for the two products and could therefore be used in conjunction with the chemical shifts in the amides to properly characterize them. Moreover, the value associated with the hydroxyl group chemical shift for violacein obtained in this study is equal to the value of 9.33 ppm reported in the study by Wille and Steglich [33].

Although it is commonly reported in the literature that deoxyviolacein is produced in modest amounts compared to violacein, the amounts produced by *Y. lipolytica* JMY7019 were equivalent to those of violacein in the 1:1:1 mixture, which is representative of a single-batch extraction. The production of both compounds and their effective purification can contribute to a potentiation of the scientific research centered around these seemingly quite polyvalent molecules. Current purification protocols with results intended for qualitative research rely on the use of flash chromatography [37,38], high-performance liquid chromatography [39], or vacuum liquid chromatography [36,40]. Compared to the present study, the purification protocols described in the literature were not provided in detail and no optimization related to the solvents used was described. While flash chromatography can offer numerous advantages over column chromatography, especially in terms of running time, it is hampered by a decreasing reliability as the amount of analyte increases [41,42], and its efficacy is highly dependent on column length and packing [41]. While these drawbacks are comparable to those of conventional column chromatography, the latter has the advantage of not relying on expensive disposable cartridges fitted into apparatuses with elevated high initial cost and non-negligible costs related to runtime and maintenance. A notable disadvantage of HPLC compared to other techniques mentioned is scale. Indeed, the method is hampered by its inability to separate compounds in significant enough volumes and high enough concentrations. In fact, the use of more concentrated analytes to mitigate these disadvantages increases the likelihood of having to deal with detrimental coelutions. Vacuum liquid column chromatography, which could help to improve the current protocol, has previously been used with a number of benign solvents including petroleum ether, ethyl acetate [36], methanol, chloroform, and n-hexane [40]. Comparatively, the solvent matrices used in these configurations are more complex than the combination used in the current study and are therefore more difficult to recycle and reuse for similar purposes.

## 3. Materials and Methods

### 3.1. Yarrowia lipolytica Strain

The *Y. lipolytica* strain JMY7019 used in this study was kindly provided by the Micalis Institute, INRAE (Jouy-en-Josas, France). Its construction has been described in an article on the extraction of violacein synthesized by this strain using anionic surfactants [22]. The working cell bank (microbial stock) was prepared as follows: a cell sample was aseptically taken from the agar plate using a sterile loop and placed in a sterile baffled flask filled with the optimized culture medium (see Section 3.2 for composition). The inoculated medium was then incubated for 24 h at 28 °C with shaking at 170 rpm stirring (Thermo Scientific MaxQ 6000, Aubervilliers, France). After 24 h of incubation, the cells were cryopreserved by adding 1 mL of the culture medium into the cryotubes (VWR, Rosny-sous-Bois, France), supplementing this volume with 1 mL of a 1:1 (*v*/*v*) glycerol/water mixture, and gently shaking the tube to homogenize the suspension. The tubes were stored at −80 °C in an ultra-low-temperature freezer (Panasonic MDF-U700VX, Couëron, France).

### 3.2. Culture Medium

The culture medium used in this study consisted of yeast nitrogen base (YNB) without amino acids or ammonium sulfate (1.7 g/L) and with dextrose (30 g/L), tryptophan (1 g/L), phenylalanine (0.2 g/L), tyrosine (16 mg/L), leucine (0.2 g/L), yeast extract (3 g/L), NH_4_Cl (1 g/L), and saline solution (1×). The composition of the 1000× concentration saline solution with a final volume of 1 L was as follows: H_3_PO_4_ 85% liquid—107 g, KCl—20 g, NaCl—20 g, MgSO_4_·7H_2_O—27 g, ZnSO_4_·7H_2_O—7.7 g, MnSO_4_·H_2_O—0.47 g, CoCl_2_·6H_2_O—0.3 g, CuSO_4_·5H_2_O—0.6 g, Na_2_MoO_4_·2H_2_O—0.094 g, H_3_BO—0.3 g, and water to obtain a total volume of 1 L. The medium was buffered with 50 mM KH_2_PO_4_/Na_2_HPO_4_, pH 6.8. All the components were prepared separately and sterilized either by autoclaving (20 min at 121 °C for the YNB, dextrose, leucine, phenylalanine, and yeast extract stock solutions) or by filtration, using 0.2 μm nylon filters for the other components to avoid their thermal degradation.

### 3.3. Pigment Production

The production of the pigments (violacein and deoxyviolacein) by *Y. lipolytica* JMY7019 was performed in two 1-L baffled Erlenmeyer flasks each containing 250 mL of culture medium, prepared as described in Section 2.2. The inoculation was performed aseptically using 2 mL of the cryogenically stored working cell bank. The cultures, performed in duplicate, were then incubated in a shaker (Thermo Scientific MaxQ 6000, Aubervilliers, France) at 28 °C with constant shaking at 170 rpm. After incubation for 168 h, the contents of the Erlenmeyer flasks were transferred to 250 mL Eppendorf centrifuge tubes and centrifuged at 4600 rpm for 15 min at 4 °C using a Heraeus Multifuge 3 S-R (Villebon-sur-Yvette, France). The supernatants were discarded, and a total of 15.4 g of wet biomass was obtained.

### 3.4. Extraction of Pigments Using a Thermoregulated Double-Jacket Vessel

A 10:1 ratio (volume (mL) to biomass weight (g)) of 96% ethanol (ThermoFisher, Illkirch, France) was used for the pigment extraction. The solvent was first poured into a 200 mL double-jacketed vessel (VRC SAS, Luçon, France) and heated to 28 °C under stirring at 200 rpm. A JULABO 1000F (Colmar, France) equipped with a DYNEO DD visual interface was used as the thermostat to maintain a constant temperature. The stirring apparatus was a Caframo BDC250 (Illkirch, France). Once the temperature was stabilized at 28 °C, the biomass was added for the pigment extraction. Samples of 1 mL were taken periodically and centrifuged for 10 min at 13,500 rpm using a VWR MicroStar (Rosny-sous-Bois, France). The supernatant was used for pigment quantification. After 1 h of extraction, the total suspension in the vessel was transferred to a 250 mL Eppendorf centrifuge tube and centrifuged at 4600 rpm for 15 min at 4 °C using a HERAEUS Multifuge 3SR (Villebon-sur-Yvette, France). The supernatant was immediately isolated and stored at 4 °C, whereas the recovered biomass was subjected to an additional extraction under the same conditions. In total, three analogous 1 h extractions were performed.

### 3.5. Pigment Quantification

The quantification of the violacein and deoxyviolacein was performed by measuring the optical density at 570 nm using a Thermo Electron Biomate 3 spectrophotometer (Illkirch, France). The value obtained was then divided by the molar attenuation coefficient for crude violacein found in the literature (10.955 g·L^−1^·cm^−1^) [43].

### 3.6. Pigment Separation Using Thin-Layer Chromatography

A series of solvent configurations were tested using thin-layer chromatography (TLC) to determine a stationary phase and a mobile phase that could be used together to separate the pigment output of the *Y. lipolytica* JMY7019. To this end, 5 µL of each extraction output, the pigment-loaded solvent, and a 1:1:1 mixture of the three extraction outputs were applied equidistantly on a 4 × 8 cm TLC plate. The order in which the samples were applied was as follows: Extraction 1, Extraction 2, Extraction 3, 1:1:1 mixture. Comparable sheets with the same samples were then placed in a 5 × 2 × 8 cm^3^ development chamber with 10 mL of solvent for a total of 3 min. The solvent combinations used are summarized in Table 2, and all the solvents were of analytical grade (Carlo Erba, Val-de-Reuil, France). Other organic solvent mixtures consisting of different combinations of cyclohexane, ethanol, methanol, ethyl acetate, and acetonitrile were evaluated, with or without the addition of triethylamine. Since the cyclohexane and ethyl acetate combinations showed the most promising results, our study focused on these two combinations.

### 3.7. Pigment Purification Using Column Chromatography

Once the separation was optimized by TLC, the pigments were purified by column chromatography. For this purpose, a 60 mL 1:1:1 *v*/*v*/*v* mixture of all the extraction outputs (pigments in 96% ethanol) was introduced into a round-bottom flask. The ethanol was partially evaporated using a Heidolph Instruments rotary evaporator system (Schwabach, Germany), and 0.5 g of silica 60 (Sigma Aldrich, Saint-Quentin Fallavier, France) was added to dry-load the pigment. The remainder of the solvent was then evaporated to dryness using the same evaporation system setup, and a homogeneously colored purple powder was obtained. To quantify the amount of adsorbed violacein and to verify that homogeneous silica coloration had occurred, three samples weighing 3.7 ± 0.18 mg were isolated from the bulk of the powder and placed in 2 mL Eppendorf tubes. Each of these tubes was filled with 1 mL of 96% ethanol and vortexed to allow a complete desorption of the pigment matrix and its diffusion into the solvent. The tubes were then centrifuged at 13,500 rpm for 10 min before spectrophotometric quantification of the supernatant crude pigment as described in Section 3.5. The average percentage of violacein in the samples was 3.3 ± 0.2%.

### 3.8. Pigment Analysis

#### 3.8.1. Pigment Analysis Using High-Performance Thin-Layer Chromatography (HPTLC)

The analysis of the violacein and deoxyviolacein in the original biosynthesized mixture was performed by HPTLC (CAMAG, Muttenz, Switzerland). This allowed the proportion of each pigment in the mixture to be determined. To this analysis, samples were first diluted in 96% ethanol and transferred to standard vials sealed with septum caps. A CAMAG ATS 4 applicator tool (CAMAG, Muttenz, Switzerland) automatically drew 2 to 10 µL of the sample and applied it to an F254 silica-covered glass plate (Merck, Saint-Quentin Fallavier, France). The plate was then transferred to a CAMAG ATC2 automatic developing chamber which was first saturated with 15 mL 65:35 ethyl acetate/cyclohexane vapor and wherein 15 mL of the same solvent was automatically poured into the bottom of the integrated glass chamber. Upon completion of the solvent migration, pigment separation was observed on the silica plate. The plate was then placed in a CAMAG TLC scanner 4 (CAMAG, Muttenz, Switzerland), which scanned the solvent fronts at a wavelength of 570 nm and processed the data to obtain the proportions of each compound in the anolyte.

#### 3.8.2. Pigment Analysis Using Nuclear Magnetic Resonance (NMR)

After purification of the violacein and deoxyviolacein, the solvent was evaporated using a rotary evaporation system, and each of the pigments was dissolved in 0.5 mL of deuterated dimethyl sulfoxide (DMSO-*d*6) prior to analysis by proton nuclear magnetic resonance (^1^H NMR) spectroscopy at 400 MHz; a BRUKER ASCEND 400 instrument was used to confirm the identity of the compounds and to verify that effective separation had occurred. The results obtained were compared with those of Wille and Steglich [33], who synthetized violacein and deoxyviolacein and confirmed the purity of the compounds using ^1^H NMR spectroscopy.

## 4. Conclusions

The literature on the separation of violacein and deoxyviolacein describes a number of methods with varying degrees of efficacy and innocuousness. The method described in this paper focuses on the use of column chromatography using silica 60 as the stationary phase and ethyl acetate/cyclohexane as the mobile phase. Compared to HPLC, our method allows for the recovery of higher amounts of pigments. The recovered products were pure as determined by ^1^H-NMR spectroscopy. The method can be optimized to determine the pigment load ceiling to maximize efficiency. These positive effects can be further enhanced by incorporating solvent recirculation through the column. In this scheme, solvents of known composition would be reintroduced into the column over multiple cycles to induce the desired effects throughout the column without the need to renew the solvent. Coupled with a larger column, increased solvent flow, and optimized for the proportions of the column, such a setup could be enabled by the use of modest amounts of solvent and silica.

## Figures and Tables

**Table 1:** Comparison between the ^1^H NMR shifts for the indolic and amide protons measured by Wille and Steglich [33], and those obtained in the current study for the confirmation of molecular identity.

Position of NH Functional Groups	Violacein		Deoxyviolacein	
	Wille and Steglich [33]	This Study	Wille and Steglich [33]	This Study
1	11.88 ppm	11.92 ppm	12.09 ppm	12.12 ppm
2	10.72 ppm	10.74 ppm	10.78 ppm	10.83 ppm
3	10.60 ppm	10.61 ppm	10.61 ppm	10.66 ppm

**Table 2:** Solvent mixtures investigated using thin-layer chromatography to be used for column chromatography.

A	Ethyl acetate/cyclohexane (10:90)	E	Ethyl acetate/cyclohexane (50:50)
B	Ethyl acetate/cyclohexane (20:80)	F	Ethyl acetate/cyclohexane (60:40)
C	Ethyl acetate/cyclohexane (30:70)	G	Ethyl acetate/cyclohexane (65:35)
D	Ethyl acetate/cyclohexane (40:60)	H	Ethyl acetate/cyclohexane (80:20)

## Data Availability

There is no supplemental data in addition to what was added in this work.
